# A Spatiotemporal Calibration Algorithm for IMU–LiDAR Navigation System Based on Similarity of Motion Trajectories

**DOI:** 10.3390/s22197637

**Published:** 2022-10-09

**Authors:** Yunhui Li, Shize Yang, Xianchao Xiu, Zhonghua Miao

**Affiliations:** School of Mechatronic Engineering and Automation, Shanghai University, Shanghai 200444, China

**Keywords:** multisensor fusion, multisensor calibration, factor graph optimization, state estimation

## Abstract

The fusion of light detection and ranging (LiDAR) and inertial measurement unit (IMU) sensing information can effectively improve the environment modeling and localization accuracy of navigation systems. To realize the spatiotemporal unification of data collected by the IMU and the LiDAR, a two-step spatiotemporal calibration method combining coarse and fine is proposed. The method mainly includes two aspects: (1) Modeling continuous-time trajectories of IMU attitude motion using B-spline basis functions; the motion of the LiDAR is estimated by using the normal distributions transform (NDT) point cloud registration algorithm, taking the Hausdorff distance between the local trajectories as the cost function and combining it with the hand–eye calibration method to solve the initial value of the spatiotemporal relationship between the two sensors’ coordinate systems, and then using the measurement data of the IMU to correct the LiDAR distortion. (2) According to the IMU preintegration, and the point, line, and plane features of the lidar point cloud, the corresponding nonlinear optimization objective function is constructed. Combined with the corrected LiDAR data and the initial value of the spatiotemporal calibration of the coordinate systems, the target is optimized under the nonlinear graph optimization framework. The rationality, accuracy, and robustness of the proposed algorithm are verified by simulation analysis and actual test experiments. The results show that the accuracy of the proposed algorithm in the spatial coordinate system relationship calibration was better than 0.08° (3δ) and 5 mm (3δ), respectively, and the time deviation calibration accuracy was better than 0.1 ms and had strong environmental adaptability. This can meet the high-precision calibration requirements of multisensor spatiotemporal parameters of field robot navigation systems.

## 1. Introduction

Multisensor fusion is an important way to aid in the environmental perception and navigation of robots in complex environments. It can fully use the advantages of different sensors, and they can then compensate each other, and achieve more accurate and robust perception and localization performance than that of a single sensor. Therefore, in recent years, it has received extensive attention, such as in driverless cars, field robots, high-precision map construction, and target tracking [1,2,3,4]. LiDAR is widely used in robot navigation due to its high measurement accuracy, insensitivity to light, and good reliability. Its fusion with the environment-independent IMU can achieve robust perception and localization in complex environments. The calibration of the spatiotemporal relationship between sensors is the premise of realizing multisensor information fusion, which mainly includes the calibration of the spatial transformation relationship between sensor coordinate systems and the calibration of the time deviation of sensor acquisition data.

To realize the calibration of the multisensor spatial transformation relationship in a robot navigation system, many methods have emerged in recent years that are mainly divided into methods based on calibration equipment and self-calibration methods. The method based on calibration equipment refers to the estimation of spatial transformation parameters using prior information provided by external sensors, rotary tables, pendulums, calibration targets, and other equipment. The calibration accuracy of this method is related to the calibration equipment [5,6,7,8]. For example, Liu et al. used a precision turntable, a standard plane, and spherical and cylindrical targets to estimate the motion of LiDAR, modeled the calibration problem as a maximal likelihood estimation problem, and solved it, but this method is time-consuming and labor-intensive, and requires expensive calibration equipment. This severely limits the scope of application of this method [9]. Since the self-calibration method does not have special calibration equipment constraints, calibration is convenient, fast, and widely popular because of its good adaptability. This is the current state of the art calibration scheme. For example, Geiger et al. proposed a motion-based calibration method, and its accuracy is related to the uncertainty of the motion estimation of the sensor to be calibrated [10]. Michael et al. constructed a camera–IMU spatiotemporal calibration problem as a nonlinear optimization problem, and solved the spatiotemporal calibration parameters by obtaining the trajectories of each sensor. This method assumes that the motion trajectory of the sensor has second-order differentiability, and needs to obtain the three-dimensional (3D) information of the environment in advance to realize the Euclidean calibration of the translation parameters [11]. Another study [12] used Gaussian process regression to model the IMU measurement, and combined the IMU preintegration method to obtain the measurement value of the LiDAR sampling time. In the probability framework, the minimal distance from the point to the plane is used as the constraint condition to construct a method. A nonlinear optimization problem solves the spatial transformation parameters, but the method requires some known planar features as a map. Furgale et al. developed the Kalibr calibration software. It is a method that jointly estimates the time deviation and the external parameters between sensors on the basis of a maximal likelihood estimation framework, and derives and realizes the estimation of the spatiotemporal parameters between multiple sensors on the basis of the B-spline basis function. This is an offline calibration method that also requires the participation of the calibration target [13]. Rehder et al. adopted the same strategy to calibrate a camera–IMU–lidar system. First, they calibrated the coordinate system transformation relationship between the camera and the IMU on the basis of continuous-time nonlinear optimization, and then calibrated the external parameters between the LiDAR and the IMU according to the calibration results [14]. Lv et al. proposed a method for calibrating the transformation parameters between LiDAR and IMU coordinate systems on the basis of B-spline continuous-time motion trajectory modeling. The B-spline curve was used to obtain IMU measurement data at the time of LiDAR sampling. The calibration parameters were solved by means of nonlinear optimization [15]. Furthermore, in recent years, methods for multisensor calibration using fuzzy logic and neural networks have also emerged. In [16], the authors proposed a method performing line feature-based factor graph optimization and producing fuzzy inference systems to adapt the covariance of the factor graph and implement the self-calibration of external parameters between multiple lidars. The authors in [17] modeled the calibration process of camera–IMU extrinsic calibration using model-free deep reinforcement learning to derive a policy that guides the motions of a robotic arm holding the sensors to efficiently produce measurements. The method automatically generates favorable and calibrated motion trajectories through the learning strategy, which can greatly improve the calibration efficiency and has high calibration accuracy.

Another problem that arises when dealing with the data fusion of multisensor systems is data synchronization. To calibrate the multisensor time offset, development has been rapid in recent years. For example, Mair et al. proposed an initialization method using cross-entropy or phase-consistent multisensor spatiotemporal calibration to separate the calibration variable from other variables and establish an analytical time deviation model. This can provide better prior information for the calibration algorithm without being affected by other variables [18]. Kelly et al. aligned the rotation curve of the camera and the IMU, and used the variant ICP method to match the rotation curve to realize the offline calibration of the time offset [19]. Li et al. proposed an online temporal offset calibration motion-estimation method based on the Multi-State Constraint Kalman Filter (MSCKF) framework for the problem of temporal offset estimation in vision–IMU systems, which has the advantage of low computational complexity [20,21]. Qin jointly optimized the time deviation, camera state, and IMU state to realize the online calibration of the time deviation between the camera and IMU data [22]. In [23], aiming at the problem of time synchronization between the camera and IMU data, combined with the extended Kalman filter state estimator, an online time deviation calibration method was proposed on the basis of improving the perspective projection model. In general, there are many calibration methods for multiple sensors, but there are relatively few unified parameters of time and space between sensors to estimate online simultaneously. On the other hand, the environment faced by robots’ autonomous operation is complex and changeable, and the unified parameters are likely to change with time, so it is urgent to develop an online self-calibration method for the spatiotemporal unified multisensor parameters.

To realize the online self-calibration of the spatiotemporal unified parameters of the multisensor fusion navigation system composed of LiDAR and IMU, a two-step calibration strategy is proposed. The method first uses the B-spline basis function to model the data collected by the IMU. Then, it solves the initial value of sensor spatiotemporal parameter calibration on the basis of the motion similarity principle and hand–eye calibration method. Lastly, within the framework of Bayesian maximal likelihood estimation, the objective function of multisensor calibration parameter optimization is constructed on the basis of IMU preintegration and lidar multifeatures, and the calibration problem is solved on the basis of graph optimization theory.

## 2. Navigation System Calibration Problem Description

**Figure 1:**
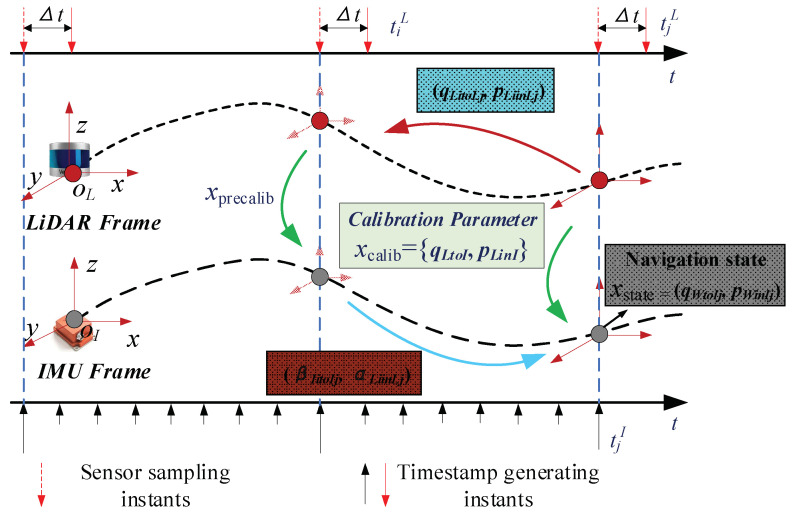
Multisensor spatiotemporal calibration problem description.

As shown in Figure 1, the robot navigation system consists of LiDAR and IMU, and the two are rigidly connected. If an arbitrary fixed coordinate system is assumed to be the world coordinate system *W*, and its *z* axis is parallel to the direction of gravity but opposite, the LiDAR coordinate system is *L*, the IMU coordinate system is *I*, the rigid body transformation relationship between the two coordinate systems is $x_{calib}$, the 3D point cloud collected by the LiDAR in time is *t*, which represents the point cloud of each frame, the angular velocity measured by the IMU in time is $\omega =\left\{\omega_{0},\cdots ,\omega_{j},\cdots ,\omega_{M}\right\}$, $\omega_{j}$ represents the angular velocity measured at different times, the gyroscope bias is $b_{g}$, the measured linear acceleration is $A=\left(a_{0},\cdots ,a_{j},\cdots ,a_{M}\right)$, $a_{j}$ represents the angular velocity measured at different times, the acceleration bias is $b_{a}$. In addition, the time deviation of the LiDAR and IMU data collection is Δt, the pose of the IMU in the world coordinate system at time $t_{j}$ is $\left\{q_{WtoI_{j}},p_{WinI_{j}}\right\}$, then the online spatiotemporal calibration of the sensors of the navigation system can be described as follows:

Estimating the state variable $X=\left(x_{calib},x_{state}\right)$ of the navigation system according to the known measurement information Z=(P,ω,A) and its initial value, and updating it dynamically, where $x_{state}=\left(q_{I_{0}toI_{0}},p_{I_{0}toI_{0}},b_{a}^{0},b_{g}^{0}\cdots ,q_{I_{N}toI_{0}},p_{I_{N}toI_{0}},b_{a}^{N},b_{g}^{N}\right)$, $x_{calib}=\left(q_{LtoI},p_{LinI},\Delta t\right)$. If the measurements conform to a Gaussian distribution and are independent of each other, the above problem description can be expressed as the following maximal posterior probability estimation form:

(1) $$\begin{array}{c} \bar{X}=\arg\max P\left(X|Z\right)=\arg\max P\left(x_{1:i}|z_{1:i}\right)\\ \;=\arg\max\prod_{i=1}^{N}P\left(x_{i}|x_{i-1}\right)P\left(z_{i-1}=z|x_{i-1}\right) \end{array}$$

where $z_{1:i}$ represents all measurement data from $t_{1}$ to $t_{i}$, $x_{1:i}$ represents all navigation system state quantities from $t_{1}$ to $t_{i}$, and when *i* = 1, set $P\left(x_{1}|x_{0}\right)=P\left(x_{1}\right)$, $P\left(z_{0}|x_{0}\right)=1$.

### 2.1. Inertial Measurement Unit Model

The inertial measurement unit is composed of a gyroscope and an accelerometer. If the angular velocity measured by the gyroscope is ${\tilde{\omega }}_{t}$ and the acceleration measured by the accelerometer is ${\tilde{a}}_{t}$, the inertial measurement unit can be modeled as follows according to the description in the literature [24]:

(2) $$\left\{ \begin{array}{l} {\tilde{\omega }}_{t}=\omega_{t}+b_{t}^{g}+n_{t}^{g}\\ {\tilde{a}}_{t}=R_{t}^{IW}\left(a_{t}-g\right)+b_{t}^{a}+n_{t}^{a} \end{array}\right.,$$

where ${\tilde{\omega }}_{t}$ and ${\tilde{a}}_{t}$ represent the measurement of the IMU at time *t*, $b_{t}^{g}$ and $b_{t}^{a}$ represent the offset of ${\tilde{\omega }}_{t}$ and ${\tilde{a}}_{t}$, respectively, $n_{t}^{g}$ and $n_{t}^{a}$ represent the white noise interference of ${\tilde{\omega }}_{t}$ and ${\tilde{a}}_{t}$, respectively, and $R_{t}^{IW}$ represent the rotation matrix from the world coordinate system to the IMU coordinate system, *g* represents the gravitational acceleration in the world coordinate system.

To use the IMU measurement data to estimate the motion of the robot body in the world coordinate system, the following dynamic model of the IMU can be established according to the IMU model:

(3) {q˙WI(t)=12Ω(ω(t))qWItp˙W(t)=vWI(t),v˙W(t)=RWItTa(t)b˙g(t)=ng,b˙a(t)=na,

where Ω(•) represents an antisymmetric matrix, and qWI represents the quaternion of the rotation matrix RWI. The pose increment can be obtained by integrating the above formula at the time interval $t\in \left[t_{k},t_{k+1}\right]$.

### 2.2. 3D LiDAR Ranging System Model

**Figure 2:**
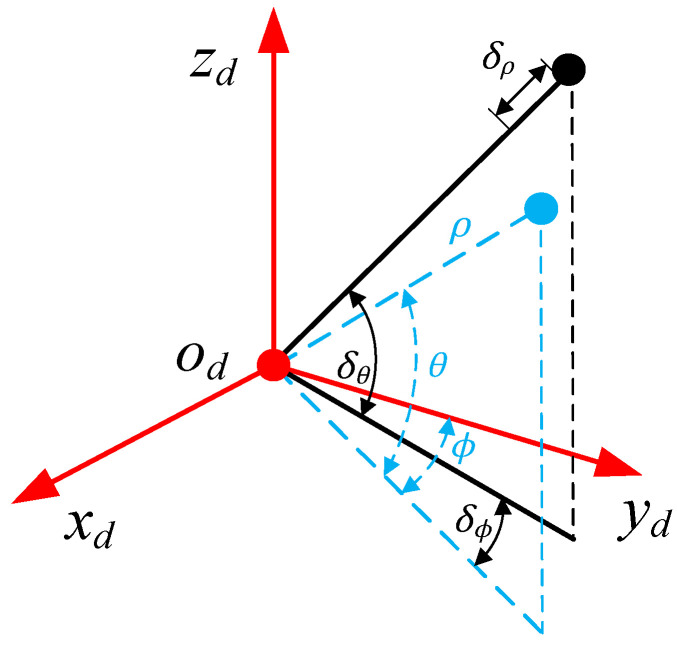
Schematic diagram of LiDAR ranging system.

As shown in Figure 2, according to the literature [25], the mechanical rotation LiDAR measurement is described as (ρ,θ,ϕ) in the spherical coordinate system; then, the measurement $\left(x_{d},y_{d},z_{d}\right)$ in the LiDAR Cartesian coordinate system can be expressed as:

(4) $$\left[\begin{array}{c} x_{d}\\ y_{d}\\ z_{d} \end{array}\right]=\left[\begin{array}{c} \rho \cos\theta \sin\phi \\ \rho \cos\theta \cos\phi \\ \rho \sin\theta \end{array}\right],$$

If the measurement error correction amount is set as $\left(\delta_{\rho },\delta_{\theta },\delta_{\phi }\right)$, the corrected LiDAR measurement can be expressed as Equation (4) in the Cartesian coordinate system. In addition, since the LiDAR measurement is the coordinate value $P\left(x_{d},y_{d},z_{d}\right)$ in the Cartesian coordinate system, it is necessary to first convert $P\left(x_{d},y_{d},z_{d}\right)$ into the spherical coordinate system, perform error compensation, and then transform it into the Cartesian coordinate system.

(5) $$\left[\begin{array}{c} {\tilde{x}}_{d}\\ {\tilde{y}}_{d}\\ {\tilde{z}}_{d} \end{array}\right]=\left[\begin{array}{c} \left(\rho +\delta_{\rho }\right)\cos\left(\delta_{\theta }\right)\sin\left(\phi -\delta \phi \right)\\ \left(\rho +\delta_{\rho }\right)\cos\left(\delta_{\theta }\right)\cos\left(\phi -\delta \phi \right)\\ \left(\rho +\delta_{\rho }\right)\sin\left(\delta_{\theta }\right) \end{array}\right],$$

### 2.3. Geometric Model of Robot Motion Trajectory

According to the literature [26], when the robot motion trajectory control nodes are uniformly distributed, the B-spline basis function in the form of the following matrix can be used for modeling:

(6) $$p\left(t\right)=\sum_{j=0}^{d}u^{T}M_{\left(j\right)}^{\left(d+1\right)}p_{i+j},$$

where *d* is the order of the B-spline basis function, which means that the continuous motion trajectory p(t) in the $t\in \left[t_{i},t_{i+1}\right)$ interval is only determined by the control point $p_{i},p_{i+1},\cdots ,p_{i+d}$ corresponding to time $t_{i},t_{i+1},\cdots ,t_{i+d}$; $u^{T}=\left[1,u,\cdots ,u^{d}\right]$, $u=\left(t-t_{i}\right)/\left(t_{i+1}-t_{i}\right)$, $M_{\left(j\right)}^{\left(d+1\right)}$ represents the *j*-th column of the spline matrix $M^{\left(d+1\right)}$; therefore, the accumulation of motion trajectories can be expressed as:

(7) $$p\left(t\right)=p_{i}+\sum_{j=1}^{d}u^{T}{\tilde{M}}_{\left(j\right)}^{\left(d+1\right)}\left(p_{i+j}-p_{i+j-1}\right),$$

The authors in [27] modeled the uniformly distributed unit quaternion control point $q_{i}$ in SO(3) space:

(8) $$q\left(t\right)=q_{i}⊗\prod_{j=1}^{d}Exp\left(u^{T}{\tilde{M}}_{\left(j\right)}^{\left(4\right)}Log\left(q_{i+j-1}^{-1}⊗q_{i+j}\right)\right),$$

where Exp(•) represents the mapping from Lie algebras to Lie groups, and Log(•) represents the mapping from Lie groups to Lie algebras.

This paper uses the cubic spline basis function to model the motion trajectory in which the spline matrix and the cumulative spline matrix can be expressed as:

(9) $$M^{\left(4\right)}=\frac{1}{6}\left[\begin{array}{cccc} 1 & 4 & 1 & 0\\ -3 & 0 & 3 & 0\\ 3 & -6 & 3 & 0\\ -1 & 3 & -3 & 1 \end{array}\right]{\tilde{M}}^{\left(4\right)}=\frac{1}{6}\left[\begin{array}{cccc} 6 & 5 & 1 & 0\\ 0 & 3 & 3 & 0\\ 0 & -3 & 3 & 0\\ 0 & 1 & -2 & 1 \end{array}\right],$$

## 3. Spatiotemporal Calibration of Multisensor Navigation System

**Figure 3:**
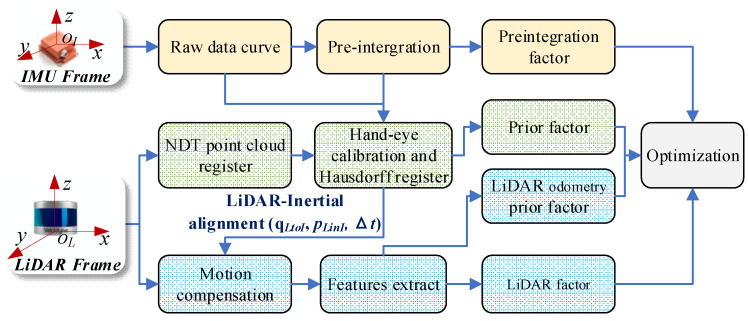
Block diagram of multisensor spatiotemporal calibration.

Since the IMU can only sense the acceleration and angular velocity information of the sensor itself, LiDAR can only measure the distance information of the surrounding environment in the sensor coordinate system. Therefore, this paper proposes a two-step spatiotemporal parameter calibration method with first coarse and then fine. First, LiDAR odometry is used. The relative motion trajectories of LiDAR are obtained by calculating, combined with the IMU motion perception information and trajectory similarity evaluation criteria, the calibration of the initial values of spatiotemporal unified parameters between IMU and LiDAR. Then, according to the model of the navigation system calibration problem in the previous section, the Bayesian factor graph model is used to construct the optimization model of the spatiotemporal calibration parameters of the navigation system, the initial value of the calibration is refined and dynamically updated, and the high-precision spatiotemporal unified parameter calibration results are obtained online. Its principle is shown in Figure 3.

### 3.1. Initial Multisensor Calibration Value Based on Local Trajectory Similarity

As shown in Figure 1, let the motion trajectory of the IMU relative to the world coordinate system be $\Gamma_{I}=\left\{q_{WtoI}\left(t_{1}\right),p_{WinI}\left(t_{1}\right)\right\}$, and the motion trajectory of the LiDAR relative to the initial position is $\Gamma_{L}=\left\{q_{L_{0}toL_{i}}\left(t_{2}\right),p_{L_{0}inL_{i}}\left(t_{2}\right)\right\}$, where $t_{1}$ and $t_{2}$ represent the time stamps of IMU data acquisition and LiDAR data acquisition time stamp, respectively. The time deviation can be expressed as $\Delta t=t_{2}-t_{1}$, $q_{WtoI}$ represents the attitude rotation matrix from the IMU coordinate system to the world coordinate system, $p_{WinI}$ represents the translation vector of the IMU coordinate system in the world coordinate system, and $q_{L_{0}toL_{i}}$ represents the rotation matrix of LiDAR coordinate system to the LiDAR initial coordinate system, $p_{L_{0}inL_{i}}$ represents the translation vector of the LiDAR coordinate system in the LiDAR zero coordinate system. If the trajectory estimation accuracy of the two rigidly connected sensors is high enough, and there is no time deviation between the sensors, then the shapes of their attitude motion trajectories $\Gamma_{I}\left(q\right)$ and $\Gamma_{L}\left(q\right)$ are similar, so points qI and qL at the same moment on the two trajectories can be arbitrarily selected to calculate the two transformation relationship between trajectories is as follows:

(10) $${\tilde{q}}_{ItoL}q_{WtoI_{i}}q_{I_{0}toW}{\tilde{q}}_{LtoI}=q_{L_{0}toL_{i}},$$

where the attitude trajectory $q_{WtoI_{i}}$ of the IMU can be obtained by solving Equation (6).

Using the transformation relationship ${\tilde{q}}_{LtoI}$ between the trajectories obtained according to Equation (8) to transform the IMU estimated motion estimation to obtain the trajectory ΓIL(q), and because the Hausdorff distance can describe the similarity between two discrete trajectories, it can be expressed according to Equation (11) as Hausdorff distance that solves the similarity between ΓIL(q) and ΓII(q).

(11) $$H_{x}=\max\left(h\left(x_{i},x_{j}\right),h\left(x_{j},x_{i}\right)\right),$$

**Figure 4:**
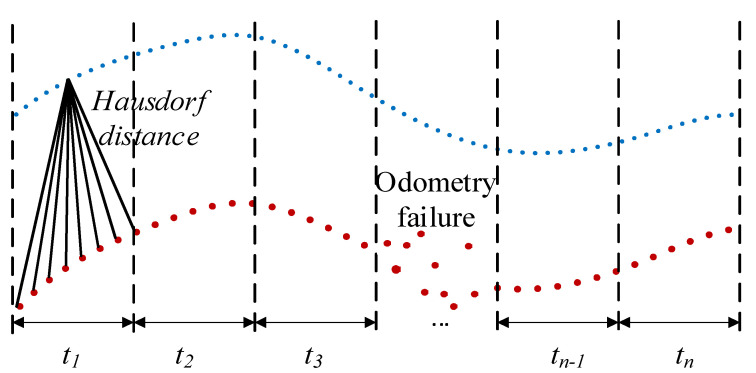
Trajectory similarity selects initial calibration trajectory segment. The red dots represent the lidar odometry localization data; the blue dots represent the IMU odometer localization data.

(12) h(xi,xj)=maxxi∈ΓIIxminxj∈ΓILx‖xi(t)−xj(t−Δt)‖h(xj,xi)=maxxj∈ΓILxminxi∈ΓIIx‖xj(t)−xi(t−Δt)‖

where *H* represents the similarity between trajectories. To eliminate the failure area of odometry estimation, the trajectory is segmented as shown in Figure 4, and the Hausdorff distance within the trajectory segment is calculated separately. If the similarity between trajectories is less than the set threshold, the estimated trajectory can be used for initial parameter calibration.

In addition, inspired by the literature [9], according to Equation (8), the angular velocity measured by the gyroscope is used to estimate the continuous time motion trajectory ΓII(t) of the IMU attitude, which can avoid the accumulation of errors caused by the measurement error on the results of the integration method, and is estimated by the NDT point cloud matching algorithm. The relative motion trajectory ΓLL of the LiDAR, and the corresponding IMU motion trajectory point is obtained according to the timestamp of each point on the LiDAR motion trajectory. Lastly, each corresponding timestamp on the two sensor trajectories is transformed according to Equation (10), and the nonlinear least-squares problem is constructed:

(13) $$f\left({\tilde{q}}_{ItoL},\Delta t\right)=ar\cos\left({\tilde{q}}_{ItoL}q_{I_{i}toI_{0}}\left(i+\Delta t\right){\tilde{q}}_{LtoI}q_{L_{0}toL_{i}}\right),$$

For all the trajectory points that meet the requirements, the cost is calculated with the above formula, the Levenberg–Marquardt (LM) nonlinear optimization algorithm is used to solve the above nonlinear least squares optimization problem, and the initial values $q_{LtoI}$ and Δt of the spatiotemporal unified parameters are obtained. After the initial parameters are obtained by calibration, the preintegration operation is used to integrate the IMU measurement, the motion at the moment of LiDAR frame data acquisition is obtained by preintegration operation, the motion distortion of LiDAR data acquisition is compensated accordingly, and the above process is repeated to obtain the final initial calibration parameters.

### 3.2. Multisensor Spatiotemporal Parameter Calibration Nonlinear Optimization

**Figure 5:**
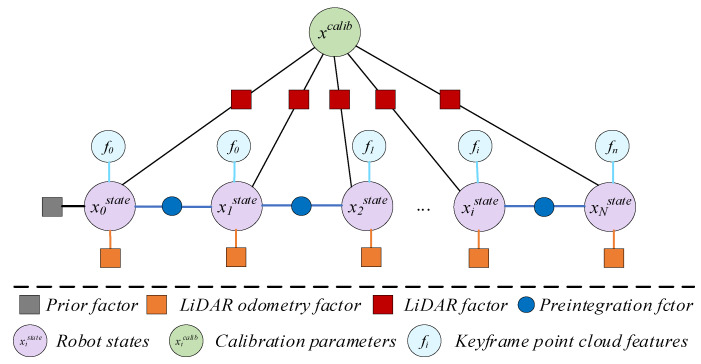
Multisensor spatiotemporal calibration factor graph model.

Since the above initial value calibration results do not consider the bias effect of the IMU, and the data acquisition frame rate of LiDAR is low, the initial value calibration accuracy of the above multisensor spatiotemporal unified parameters is still insufficient. Therefore, in this section, the initial calibration results of external parameters as the initial value, according to the description of the state estimation problem in this paper by Equation (1), use the factor graph model to model it, fully using the sensor motion sensing information of the navigation system, and building the factor graph model shown in Figure 5, which mainly includes: prior factor, IMU preintegration factor, LiDAR factor, LiDAR odometer prior factor. The nonlinear optimization algorithm is used to solve the above model to obtain high-precision calibration results.

#### 3.2.1. IMU Preintegration Factor

To avoid the repeated integration problem after the linearization point changes, the literature [28] proposed a preintegration method to integrate the motion state in the interval. According to the preintegration formula, this paper uses the preintegration amount between the two states of the IMU as the constraint factor to constrain the navigation state between the two moments:

(14) $$\left\{ \begin{array}{l} e_{p}^{w}=q_{b_{i}w}\left(p_{wb_{j}}-p_{wb_{i}}-v_{i}^{w}\Delta t+\frac{1}{2}g^{w}\Delta t^{2}\right)-\alpha_{b_{i}b_{j}}\\ e_{q}^{w}=2{\left[q_{b_{j}b_{i}}⊗\left(q_{b_{i}w}⊗q_{wb_{j}}\right)\right]}_{xyz}\\ e_{v}^{w}=q_{b_{i}w}\left(v_{j}^{w}-v_{i}^{w}+g^{w}\Delta t\right)-\beta_{b_{i}b_{j}}\\ e_{b_{a}}^{w}=b_{j}^{a}-b_{i}^{a}\\ e_{b_{g}}^{w}=b_{j}^{g}-b_{i}^{g} \end{array}\right.,$$

where $q_{bw}$ and $p_{wb_{j}}$ represent the attitude transformation quaternion and translation vector between the world coordinate system and the IMU coordinate system, respectively; $v^{w}$ represents the velocity vector of the IMU coordinate system in the world coordinate system; $g^{w}$ represents the gravitational acceleration in the world coordinate system; and $b_{a}$ and $b_{g}$ represent the accelerometer and gyroscope bias errors, respectively.

#### 3.2.2. LiDAR Odometry Prior Factor

Because LiDAR odometry was used to estimate the motion state $T_{L_{0}toL_{i}}^{}$ of the unmanned system in the initial calibration stage of external parameters, in order to fully use the LiDAR point cloud information to estimate the motion state of the unmanned system and the external structure parameters of the sensor, the LiDAR odometer is introduced in each frame to firstly estimate the motion state *T* of the unmanned system. In addition, considering that the point cloud collected by LiDAR is seriously distorted when the unmanned system moves violently, the accuracy of the above NDT-based LiDAR odometer is low. Therefore, this paper uses the B-spline interpolation algorithm to process the above preintegrated data. Solve the pose of the LiDAR coordinate system relative to the LiDAR initial zero coordinate system (defined as the map coordinate system) at the time of LiDAR point collection.

(15) $$P_{i+\tau }^{L_{0}}=T_{LtoI}^{-1}\cdot T_{WtoI_{0}}\cdot T_{WtoI_{i}}^{-1}\cdot T_{I_{i}toI_{i+\tau }}^{-1}\cdot T_{LtoI}P_{i+\tau }^{L_{i+\tau }},$$

where $T_{LtoI}$ represents the external parameters between sensors; $T_{WtoI_{0}}$ represents the pose of the IMU zero coordinate system in the world coordinate system; $T_{WtoI_{i}}$ represents the pose of the IMU coordinate system in the world coordinate system at time $t_{i}$; $T_{I_{i}toI_{i+\tau }}$ represents the IMU pose of the LiDAR point sampling time between $t_{i+\tau }$ and $t_{i}$ that can be obtained with the interpolation of Equations (7) and (8). $T_{L_{0}toL_{i}}^{}$ is recalculated after removing the distortion.

#### 3.2.3. LiDAR Odometry Factor

1. Point Cloud Feature Extraction;

To extract the features of 3D point clouds, in 1999, Mass, Vosselman, et al. proposed to use the invariant spatial moment of the point cloud to represent the geometric characteristics of the point cloud [29]. In 2009, Jutzi, Cross, et al. used a 3D covariance matrix (i.e., the 3D structure tensor) composed of the invariant moments of the point cloud to describe the geometric features of the 3D point cloud in this range [30]. This paper uses the structure tensor to extract the geometric features of the point cloud. It can be expressed as follows.

First, the 3D structure tensor (covariance matrix) composed of point cloud spatial moment is calculated.

(16) $$S=\frac{1}{k}\sum_{i\in P}\left(p_{i}-\bar{p}\right){\left(p_{i}-\bar{p}\right)}^{T},$$

where *P* and $\bar{p}$ represent the k-nearest neighbor set and the center point of the set *P*, respectively.

Second, matrix decomposition is performed on the structure tensor to obtain eigenvalues and eigenvectors.

(17) $$S=U\Sigma V^{T},\Sigma =diag\left(\lambda_{1},\lambda_{2},\lambda_{3}\right),$$

Lastly, compute geometric description features from eigenvalues.

(18) $$\mathrm{linearity}:\frac{\lambda_{1}-\lambda_{2}}{\lambda_{1}},\mathrm{planarity}:\frac{\lambda_{2}-\lambda_{3}}{\lambda_{1}},$$

Only point clouds whose linearity and planarity are within a certain threshold range are retained as the extracted line features and plane features. On the other hand, considering the sparseness of LiDAR point cloud, this paper extracts point cloud features of points, lines, and planes in the environment according to the method of the literature [31].

2. LiDAR Factor;

First, the invariant moment of the 3D point cloud within a certain range is calculated for each frame of point cloud, and the covariance matrix $\Sigma =diag\left(\lambda_{1},\lambda_{2},\lambda_{3}\right)$ is constructed to describe the geometric characteristics of the 3D point cloud within this range. The geometric description features of the point cloud are obtained, and the point, line, and plane features are extracted accordingly. In addition, planar features are extracted by evaluating the curvature of adjacent points, and points with small curvature values are classified as planar features. Cross-validation checks are performed on the geometric features extracted by the above methods to obtain the final point, line, and plane features. The following constraint factors are constructed according to the distance between point-to-point, line, and plane:

(19) {epo→po=qMi−(RLitoMpi+tLiinM),qMi,pi∈Fi+1poepo→li=vMj×(qMj−(RLitoMpj+tLiinM)),qMi,pi∈Fi+1liepo→pl=nMkT(qMk−(RLitoMpk+tLiinM)),qMk,pk∈Fi+1pl,

where $e^{po\to po}$, $e^{po\to li}$, and $e^{po\to pl}$ represent the distance between points, point-to-lines, and point-to-planes, respectively; and vMj and nMk represent the direction vector and normal vector in the feature line and plane in the map coordinate system, respectively. $\left[R_{L_{i}toM},t_{L_{i}inM}\right]$ represents the pose between the current LiDAR coordinate system and the map coordinate system.

#### 3.2.4. Objective Function of Nonlinear Optimization

To realize the unified parameter calibration of sensor data, under the conditional of the measurement noise and the process noise conform to Gaussian distribution, the following nonlinear optimization problem is constructed:

(20) $$\begin{array}{c} \underset{X}{\min}\left\{E_{INS}^{prior}+\sum_{i\in I}\lambda_{lo}E_{i}^{lo}+\sum_{\left(i,i+1\right)\in B}\lambda_{imu}{\left\|e^{imu}\right\|}_{\Sigma_{e_{i}^{imu}}}^{2}+\sum_{p\in F_{i}}\lambda^{po,pl,li}{\left\|e_{i}^{po\to po,pl,li}\right\|}_{\Sigma_{e_{i}^{po,pi,li}}}^{2}\right\} \end{array},$$

where $E_{INS}^{prior}$ is the system prior information, $E_{i}^{lo}$ is the LiDAR odometry prior factor; $\Sigma_{e_{i}^{\cdot }}$ is the covariance matrix of the factor, and $\lambda_{\left(•\right)}$ is the adjustment parameter ($3\times N\times M^{-1}\times mean\left(\Sigma_{e_{i}^{po,pl,li}}^{-1}\right)\times mean\left(tr\left(\Sigma_{e_{•}^{imu}}\right)\right)$). The proposed algorithm is as follows (Algorithm 1).
**Algorithm1:** LiDAR/IMU spatiotemporal calibration.**Input:** IMU set ***I*** [1], point cloud set ***P*** {***P*_1_**, …, ***P*_N_**} **Output:** External spatial parameter ***T***_il,_ temporal parameter Δ*t* **while** (True) **do** Modeling Gyro measurement data set ***I*_i_** with B-splines Solving the motion between lidar frames by NDT algorithm Solving external parameters ***T***_il0_ using hand–eye calibration Time offset Δ*t_0_* solving to minimize the Hausdorff distance Preintegration using IMU measurement **for** *i* = 1, …, *K*
**do** Distortion correction of point cloud by initial calibration    Construct IMU preintegration factor, lidar prior factor and lidar factor    Perform nonlinear optimization on the data in the window to solve the state ***x****_i_* **  end** **end**

## 4. Simulation Analysis

**Figure 6:**
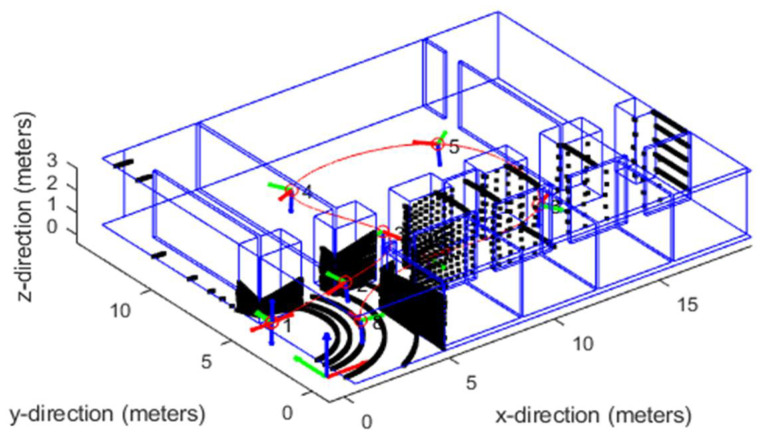
Schematic diagram of the trajectory of IMU and LiDAR.

To verify the feasibility and statistical properties of the algorithm, the following simulation experiments were designed in which the sampling frequency of IMU was 200 Hz, the sampling frequency of LiDAR was 10 Hz, the number of LiDAR lines was 16, the resolution of horizontal angle was 0.25°, the resolution of vertical angle was 2°, and the gravitational acceleration vector was (0, 0, 9.81) m/s^2^. The noise of the sensor was simulated with Gaussian white noise in which the LiDAR point cloud noise conformed to the mean value of 0, variance of 2 cm, the bias of the gyroscope was 10^−5^, the bias of the acceleration was 10^−4^, the error of the gyroscope was 0.00015, and the error of the acceleration was 0.00019. The simulation environment is shown in Figure 6. The plane height in the environment was 3 m, the simulation time was 123 s, and the stabilization time of 3 s was set at the start time. The rotation angle relationship between lidar and IMU was (*Ax*, *Ay*, *Az*) = (0,180,0)/°, and the translation relationship was (*Tx*, *Ty*, *Tz*) = (0, 40, −60)/mm. The motion trajectory was obtained by cubic spline interpolation as shown in Table 1 for the control point data. The simulation of time offset is realized by translating the timestamps of LiDAR trajectory by 5, 10, 15, 20, and 30 ms. All simulations were performed on a computer with Intel core i7-9700K and 32 GB memory using MATLAB 2019 b as the simulation tool.

**Figure 7:**
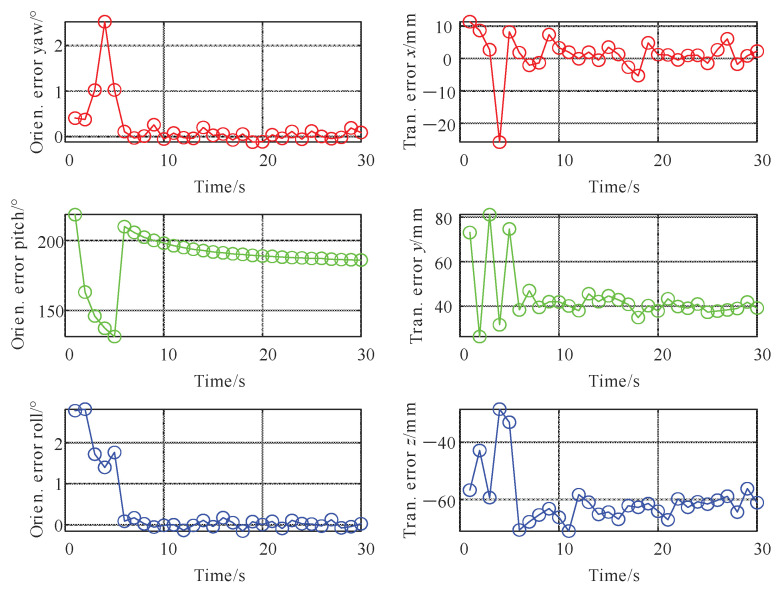
External parameters’ calibration error of IMU and LiDAR varies with time.

Under the above simulation parameter settings [13], Kalibr and the algorithm proposed in this paper were used to solve the spatial unified parameters. The Kalibr algorithm is an offline solution after importing the LiDAR data; statistics of the calibration error of its spatial parameters are shown in Figure 7, and the calibration error of time deviation is shown in Table 2.

Figure 6 shows that the translation calibration error of the algorithm proposed in this paper fluctuated greatly within the initial 5 s. This was mainly because the addition time interval of the initial calibration parameters is set to 5 s. During this period, the prior calibration factor and LiDAR odometry factors dominated; however, with the increase in time, new factors were continuously added, the error continued to decrease, and it basically converged after 15 s, which shows that the algorithm proposed in this paper can effectively realize the spatial parameters calibration between IMU and LiDAR.

**Figure 8:**
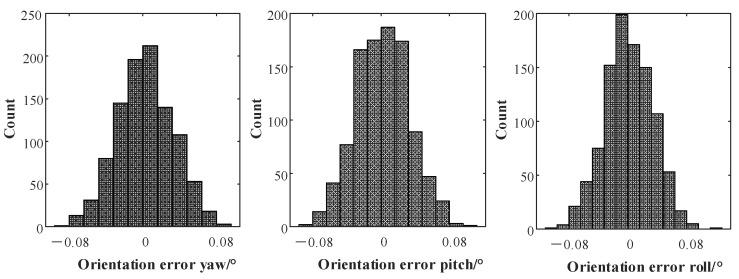

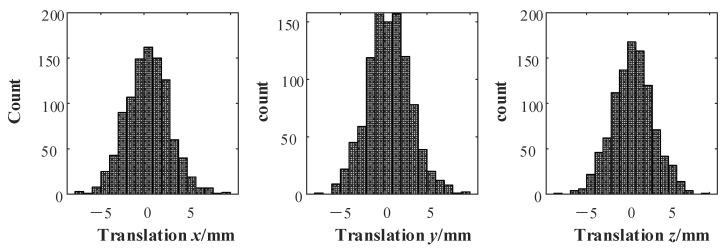
Statistical histogram of calibration errors of IMU and LiDAR external parameters.

Table 2 shows that the time deviation calibration error was significantly reduced before and after the nonlinear optimization. The optimized error is comparable to the Kalibr calibration result, and the final error was less than 0.1 ms. When the time deviation was larger, the calibration effect is better than that of Kalibr. This shows that the algorithm proposed in this paper can effectively realize the time deviation calibration between sensors. In addition, to verify the statistical characteristics of the algorithm proposed in this paper, 1000 simulation calibration experiments were performed, and the root mean square (RMS) error of each experiment was counted. The results are shown in Figure 8.

According to Figure 8, under the conditions that the sensor motion was smooth and the motion excitation was sufficient, the translation calibration error of the algorithm proposed in this paper was 5 mm (3δ), the rotation calibration error was 0.08° (3δ), and the statistical distribution of the calibration error is basically conforming to the normal distribution. This shows that the algorithm proposed in this paper can achieve accurate calibration of the external parameters of IMU and LiDAR in a statistical sense.

## 5. Experiment

**Figure 9:**
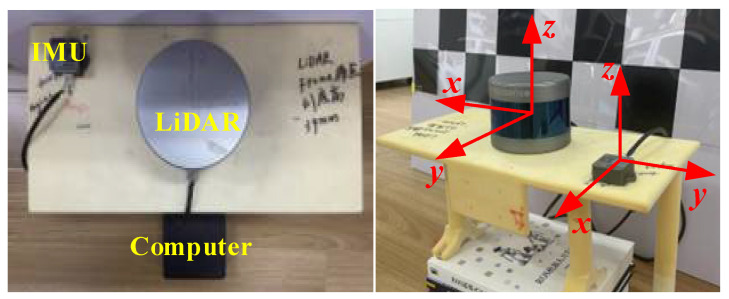
Physical map of IMU and LiDAR assembly.

To verify the algorithm proposed in this paper, an actual performance test experiment was carried out. As shown in Figure 9, in the actual multisensor external parameter calibration test, the RS-LiDAR-16 (RoboSense, Shenzhen, China) lidar was used, which is a 16-line mechanical rotating radar, and the measurement error was less than 2 cm; the inertial measurement unit adopted HandsFree HFI-A9 (HandsFree Robot, Shenzhen, China), the static angle accuracy was 0.3°, the dynamic angle accuracy was 1.5°, the acceleration range was ±8 g, the two were fixed on the same rigid body, and the data processing computer was an Intel NUC11TNK i7 (Ubuntu 20.04, Intel, Santa Clara, CA, United States) with 16 GB memory. In the actual test, the handheld sensor unit moved in 3D space to fully stimulate the acceleration and angular velocity components on each coordinate axis. At the same time, the algorithm proposed in this paper was used to solve the external parameters and time deviation between LiDAR and IMU, and it was compared with the current state of the art. Lastly, the calibrated localization and navigation unit was installed on an unmanned vehicle for localization and mapping experiments.

### 5.1. Sensor Spatiotemporal Unified Parameter Calibration Experiment

**Figure 10:**
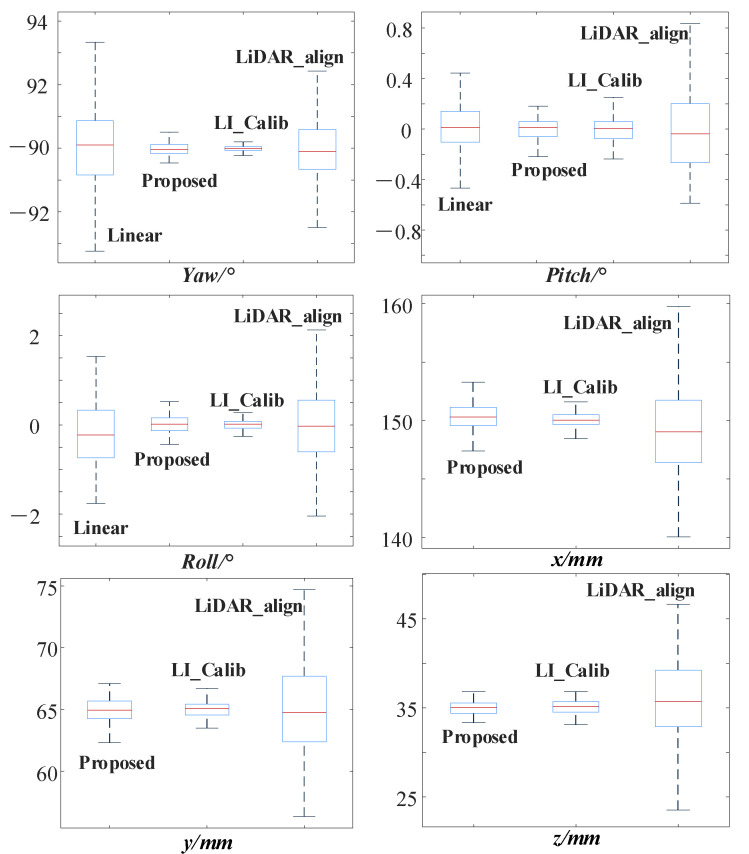
Calibration results of external parameters of IMU and LiDAR.

According to the data collected with the above-mentioned localization and navigation sensor unit, calibration tools lidar_align and calib_lidar_imu, and calibration algorithms LI-Calib and the linear and nonlinear optimization calibration algorithm proposed in this paper were used to calibrate the external parameters and time deviation. The RMS error statistics are shown in Table 3 and Figure 10, where the reference value of spatial calibration is the CAD reference value, and the reference value of time deviation is the average value of multiple calibrations as the reference value. This was segmented and then calibrated using the above algorithm for each data segment, 10 sets of calibration data were obtained, and statistics were performed.

The statistics of the spatiotemporal calibration results shown in Figure 10 and Table 3 show the following. (1) The proposed algorithm was improved compared with other calibration algorithms, and the accuracy was comparable to that of the LI-Calib calibration algorithm. The actual calibration accuracy of the proposed algorithm could reach 0.1°, 8 mm, which could meet the high-precision requirements of the unmanned system for the localization and mapping. In addition, benefiting from the algorithm proposed in this paper, the degradation of the localization and determined by considering the localization performance of the odometer during the initial calibration, and the factor graph optimization at the back end enabled the calibration algorithm to adaptively select the localization trajectory data that met the requirements of the odometer localization performance to solve the calibration parameters. (2) The error of the linear calibration algorithm was larger than that of the nonlinear optimization algorithm, and the calibration result distribution range was large compared with that of the optimization-based method. This may have been due to the continuous addition of new calibration constraints during the nonlinear optimization process, so that the calibration results converged to their true values. (3) According to the statistical results of time deviation calibration, both the nonlinear graph optimization algorithm proposed in this paper and the initial calibration algorithm could realize the calibration of time deviation. The square root error reached 0.26 ms, and the improvement in accuracy compared to the initial calibration may have been due to the back-end optimization processing the trajectory data for a longer time.

### 5.2. Sensor Fusion Localization and Mapping Test Experiment

**Figure 11:**
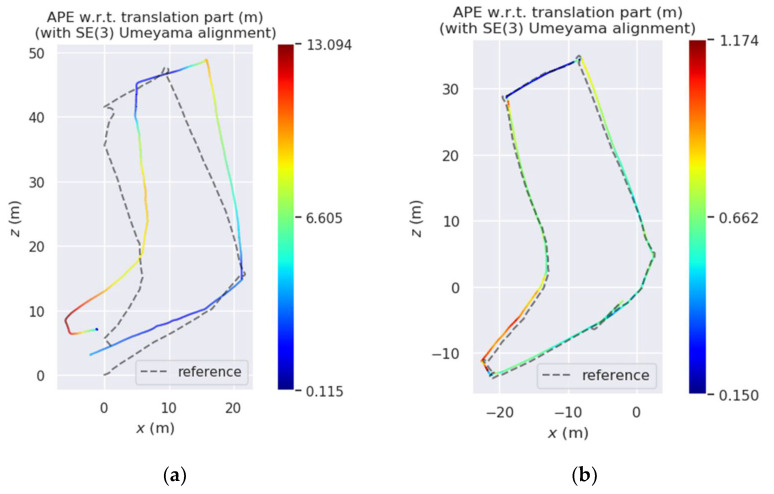
IMU and lidar odometer localization results. (**a**) LiDAR_IMU_calib; (**b**) proposed.

**Figure 12:**
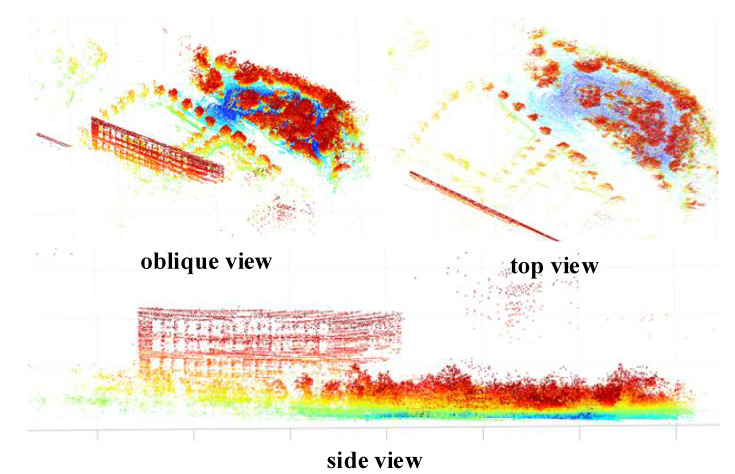
LiDAR point cloud mapping results.

To verify the calibration results obtained by the algorithm in this paper, the calibration results obtained with the calibration algorithm in this paper were combined with those of the LIO-SAM open-source algorithm and its recommended external parameter calibration algorithm to realize the odometry localization experimental environment mapping experiment of LiDAR and IMU fusion, respectively. EVO was used to evaluate the localization results as shown in Figure 11 and Figure 12 [32].

As shown in Figure 11, the localization results show that the localization accuracy was significantly improved before and after adding the spatiotemporal calibration results proposed in this paper, especially at the corners. The reasons may be as follows: (1) With the more data collected, there was an increasing number of constraints involved in calibration and motion state estimation, so that the accuracy of optimization and calibration parameters was continuously improved; (2) the calibration results and the high-frequency measurement information of the IMU could be fully used to correct the distortion of the LiDAR point cloud frame, so that the point cloud frame motion estimation was more accurate, thereby reducing distortion during violent movements such as cornering. As shown in Figure 12, from the top and side views of the environment modeling results, the point cloud map reproduced the actual scene well in both the vertical and the horizontal directions because the sensor used by the algorithm proposed in this paper is used in the environment modeling. The calibration parameters between IMU and LiDAR data were fused to achieve more accurate motion estimation, so the keyframe point cloud map stitching based on motion estimation was also more accurate.

## 6. Conclusions

Aiming at the problem of unified spatiotemporal parameter calibration between LiDAR and IMU, a spatiotemporal unified parameter calibration algorithm between sensors based on the coarse and fine combination was proposed. The similarity was measured; the goal was to minimize the similarity distance, and nonlinear optimization was performed to solve the initial calibration results. At the same time, it was fully considered that the sensor parameters are likely to change continuously during the operation of the actual unmanned system, so the calibration parameters were introduced into the factor graph of motion estimation to estimate the calibration parameters in real time. The simulation and actual test results show that the two-step calibration strategy in this paper can effectively solve the calibration parameters; because of the factor graph optimization method, it can be flexibly extended to more sensor parameter calibration and fusion localization. Furthermore, compared with the state-of-the-art LiDAR and IMU calibration method LI_Calib, the spatiotemporal online calibration method proposed in this paper can effectively eliminate odometer faults or drift, and obtain the calibration of time deviation while obtaining the calibration results with approximate accuracy.

The algorithm proposed in this paper needs to fully excite each coordinate axis of the sensor during the calibration process, so it is not suitable for unmanned systems that move on flat ground. In the follow-up, the multisensor online calibration problem on flat ground will be studied.

## Figures and Tables

**Table 1:** Motion trajectory generation control point parameters.

Num	*x/m*	*y/m*	*z/m*	*roll/*°	*pitch/*°	*yaw/*°
0	0.305	3.810	0.610	0	−180	0
1	3.810	3.810	1.219	0	−188	8
2	7.010	5.669	1.524	0	−174	95
3	7.224	11.582	0.610	0	−176	25
4	13.472	10.668	0.914	0	−185	−55
5	13.259	4.145	1.219	0	−180	−150
6	7.772	3.810	0.914	0	−180	−180
7	2.438	1.067	1.219	0	−188	−100

**Table 2:** IMU and lidar time offset calibration results.

Num.	5 ms	10 ms	15 ms	20 ms	30 ms
Kalibr	4.87	9.84	15.22	19.84	29.88
Linear *	5.19	10.21	14.81	20.18	30.15
Proposed	5.34	10.19	14.83	19.85	29.91

* The linear method refers to the time deviation initial value solution algorithm proposed in this paper.

**Table 3:** Time deviation calibration result (unit: ms).

Method	Mean	Median	Std. Dev.	RMS
Linear *	0.5993	0.6088	0.2340	0.6420
Proposed	0.2442	0.2484	0.1040	0.2647

* The linear method refers to the time deviation initial value solution algorithm proposed in this paper.

## Data Availability

Not applicable.
